# Glutathione, glutathione peroxidase and some hematological parameters of HIV-seropositive subjects attending clinic in University of Calabar teaching hospital, Calabar, Nigeria

**DOI:** 10.1186/s12879-019-4562-6

**Published:** 2019-11-08

**Authors:** Stephen Bassey Coco-Bassey, Enosakhare A. Asemota, Henshaw Uchechi Okoroiwu, Joyce E. Etura, Esienanwan Esien Efiong, Imeobong J. Inyang, Emmanuel K. Uko

**Affiliations:** 1 Haematology Unit, University of Calabar Teaching Hospital, Calabar, Nigeria; 2 Haematology Unit, Department of Medical Laboratory Science, University of Calabar, Calabar, Nigeria; 3 Department of Biochemistry, Federal University of Lafia, Nasarawa, Nigeria

**Keywords:** Antioxidants, Glutathione, Glutathione peroxidase, HIV, Calabar

## Abstract

**Background:**

Despite the numerous intervention programmes, HIV still remains a public health concern with a high impact in Sub-Saharan Africa region. Oxidative stress has been documented in HIV subjects as viral infection promotes prolonged activation of immune system, hence, production of increased reactive oxygen species.

**Methods:**

We studied 180 subjects. Of these, 60 were HIV-infected on antiretroviral therapy (ART), 40 were ART naïve HIV-infected and 80 were apparent healthy non HIV-infected subjects. The complete blood count was performed by automated hemoanalyzer, the CD4^+^ T-cell count was performed by cyflow cytometer, while the antioxidant assay was performed using ELISA technique.

**Result:**

All evaluated parameters; glutathione (GSH), glutathione peroxidase (GPX), CD4^+^ T-cell count, haemoglobin (Hb), total white blood cell count (WBC) and platelet count were significantly (*P* < 0.05) reduced in the HIV-infected subjects. All assessed parameters were found to be significantly (*P* < 0.5) reduced in the HIV-infected subjects that are ART naive when compared with those on ART. HIV-infected subjects with CD4^+^ T-cell count < 200 cells/mm^3^ had significantly (*P* < 0.05) reduced values in all assessed parameters when compared to those with CD4^+^ T-cell count ≥200 cells/mm^3^. GSH and WBC were found to be significantly (*P* < 0.05) increased in the female HIV-infected subjects when compared with the male counterpart. Anemia prevalence of 74 and 33% were recorded for the HIV-infected and control subjects, respectively. Gender and ART treatment were found to be associated with anemia in HIV. Male HIV-infected subjects on ART were found to be more likely to have anemia.

**Conclusion:**

Antioxidants; GSH and GPX were found to be significantly reduced in HIV infection. Further probe showed that the antioxidant status was improved in the HIV-infected group on ART.

## Background

There are about 36.7 million persons living with HIV as at the end of 2016. World Health Organization report estimated approximately 0.8% of adults within the age bracket of 15–45 years globally are living with HIV. Though HIV epidemic varies per region, the sub-Saharan region maintains the highest incidence such that approximately 1 in every 25 adults (4.2%) is infected by HIV giving rise to almost two-third of the population of those living with HIV globally [1, 2]. Despite the advance in virological and clinical management, HIV infection has developed into a worldwide pandemic [3]. Infection with Human Immune Deficiency Virus (HIV) is associated with progressive loss of cellular immunity which results to life-threatening opportunistic infections and progressive development of Acquired Immune Deficiency Syndrome [4]. Of all the people living with HIV globally, 9% of them live in Nigeria according to 2014 Gap report [5]. There were 3.2 million people living with HIV in Nigeria in 2016, among whom 30% were assessing antiretroviral treatment or prophylaxis to prevent transmission to their children [6]. Hence, Nigeria is now the second largest HIV disease burden in the world after South Africa which has 7.1 million (19% of global epidemic) burden of the disease, though prevalence is stable at 3.4% [7, 8]. By states, HIV prevalence in Nigeria varies. The highest number of HIV prevalence were recorded in Benue, Anambra, Bayelsa and Akwa Ibom States of the federation and the Federal Capital territory (FCT) with prevalence greater than 8%. The least HIV prevalence areas are Kebbi, Ekiti, Katsina, Jigawa and Bauchi States of the federation with prevalence range of 1–2% [8].

In 2016, Nigeria established its guideline for ART treatment [9]. However, the World Health Organization (WHO) recommendation of ART for low and middle income countries is viz.: First line ART includes 2NRTIs (1 of TDF, AZT, ABC +  1 of 3TC, FTC) +  1NNRTI (NVP or EFV) with specific recommendations for patients aged 3–10 years and patients with impaired renal function. Second line ART includes 2NRTIs (1 of TDF, AZT, ABC + 1 of 3TC, FTC) + 1PI/r (LPV/r, ATV/r) with specific recommendation for children below 3. Third line includes 1–2 NRTIs + DRV/r + 1 INI (DTG or RAL) with specific recommendations for children below 10 and pregnant women [10].

There have been wide documentation of oxidative stress in persons living with HIV infection as the infection encourages long term activation of the immune system that eventually contribute to increased production of reactive oxygen species [11–14]. Oxidative stress results from an imbalance between formation and neutralization of pro-oxidants [15]. Several lines of evidence suggest that enhanced oxidative stress also plays a pathogenic role in human immunodeficiency virus (HIV) infection [15–17]. Increase in oxidative stress may accelerate disease progression, increasing HIV replication through signalling of nuclear factor (NF)-KB as well as mutation rate of the viral RNA genome, leading to greater damage to the host [13, 14].

There are several enzymatic cum non-enzymatic antioxidants in cells that prevent or in some cases cushion damages associated with reactive oxygen species [18]. The immune system consists of many compound with varying properties: macromolecules such as albumin and ceruloplasmin; enzymes such as glutathione peroxidase, superoxide dismutase, catalase; and small molecules such as reduced glutathione, vitamin C, vitamin E, uric acid, and β-Carotene [19]. Glutathione is documented as the most copious non-protein thiol in living organisms and plays many relevant biological roles among which are: T and B cell differentiation, cell defense and cytotoxic T-cell activation. More so, it plays antioxidant role scavenging reactive oxygen species and limiting hydrogen peroxide [20]. Glutathione is a tripeptide consisting of cysteine REPLACE WITH: (L-γ-glutamyl-L-cysteinyl-glycine) that is present in minute (millimolar) quantity in all animal cells [21]. De novo*,* Glutathione (GSH) is produced from the tripeptide consisting of glycine, amino glutamine and cysteine. Glutathione occurs in two forms intracellularly; the oxidized form (GSSG) and the reduced form (rGSH). There are basically two steps involved in the formation of the rGSH which involves two enzymes; glutamate-cysteine ligase (GCL) and glutathione synthase (GSS). The first as well as the rate limiting step reaction involving the synthesis of GSH is catalysed by GCL. GCL is composed of catalytic (GCLC) and a modifier (GCLM) subunit [22, 23]. An alternative pathway is the reconversion of GSSG back to GSH in the presence of the enzyme glutathione reductase (GSR) using NADPH as cofactor [22].

This study is aimed at investigating the antioxidant status of HIV positive subjects with the view of providing information on improving the management of HIV patients.

## Method

### Study designs and subjects

A total of one hundred and eighty (180) subjects were recruited for this study. Sixty (60) of these were HIV-infected subjects on antiretroviral therapy (ART), while 40 were HIV-infected ART naive subjects. Eighty (80) apparently healthy HIV negative subjects were recruited as control. The HIV infected subjects were attending University of Calabar Teaching Hospital HIV Clinic. All subjects were drawn from both gender and aged between 18 and 60 years and were all resident in Calabar, Nigeria. Ethical approval was obtained from Health Research Ethical Committee (HREC) of the University of Calabar Teaching Hospital. Informed consent was obtained from the subjects involved in this study. Pregnant women, tuberculosis patients and those currently undergoing treatment for any chronic illness were excluded from the study. The study took place from March to April, 2016. The ART combination given to patients at the study area as at the time of study are combination of two nucleotide reverse transcriptase inhibitors and one non-nucleotide reverse transcriptase inhibitor (Tenofovir+Lamivudine+Efavirenz or Lamivudine+Zidovudine+Nevirapine). All sample assays were performed in duplicate and the average values recorded.

### Sample collection

Six milliliter (6 mL) of venous blood was collected from the subjects. Two Milliliter (2 mL) of the collected blood was dispensed into plain tube for serum harvesting for glutathione and glutathione peroxidase and the remaining 4 mL was dispensed into dipotassium ethylene diamine tetra - acetic acid (K_2_EDTA) container for complete blood count and CD4^+^ T-Cell count. The CD4^+^ T-cell count and complete blood count were analyzed immediately while the sera for antioxidant analysis were frozen at -20^o^c and analyzed within 1 week.

### Complete blood count and CD4^+^ T-cell assay

The CD4^+^ T-cell count was performed using Partex Cyflow cytometer by Partec cyflow, Germany. Following the booting of the machine, 20 μL of CD4^+^ T-cell count phycoerythrin monoclonal antibody (PEmAb) was added to a Rohren tube and subsequently by 20 μL of well mixed EDTA anticoagulated blood sample. The mixture was mixed and incubated in the dark for 15 min at room temperature. Next, 800 μL of the CD4^+^ T-cell count buffer was added. The final mixture was then mixed, plugged in the sample port, and read via the cyflow. Sysmex KX-21 N by Sysmex Corporation Kobe, Japan, was used in analysis of complete blood count. This was done following the manufacturer’s instructions.

### Antioxidant assay

The glutathione assay was performed using ELISA test kits from USCN Life Science, USA. The assay technique uses the competitive inhibition enzyme immunoassay principle in which monoclonal antibody specific to glutathione has been pre-coated onto a microplate and a competitive inhibition reaction launched between biotin labeled GSH and unlabeled GSH with the pre-coated antibody specific to GSH. Following incubation, the unbound conjugate was washed off and avidin conjugated to Horseradish peroxidase (HRP) added to each microplate well and incubated. The amount of bound HRP conjugate is inversely proportional to the concentration of glutathione present in the sample. The change is measured spectrophotometrically at 450 nm ± 10 nm.

The glutathione peroxidase (GPX) activity was performed using ELISA kits from USCN Life Science, USA. A monoclonal antibody specific to GPX (glutathione peroxidase 1) was pre-coated on the microplate. Avidin conjugated to Horseradish peroxidase (HRP) was then added to each microplate well and incubated after tetramethylbenzydine (TMB): substrate solution was added to only those well that contain GPX, biotin-conjugated antibody and enzyme-conjugated avidin will exhibit a change in colour. The enzyme-substrate reaction was terminated using the stop solution (sulphoric acid solution) and the change is measured spectrometrically at a wavelength of 450 nm ± 10 nm.

### Statistical analysis

Data were analyzed using SPSS version 20 (SPSS Inc., Chicago, USA). Categorical data were represented with frequency and percentages while continuous data were expressed as mean and standard deviations. One sample Kolmogorov-Smirnov test was used to assess the normality of the data. All data were normally distributed, hence, parametric procedure was used. Comparison of the antioxidant variable and complete blood count between the test and control were performed using independent t-test while comparison among various age groups were analyzed using ANOVA. Association between variables were analyzed using Chi Square and Fischer exact test. Risk estimates were analysed using odd ratio. Alpha value of 0.5 was used.

## Result

The average age of the HIV-infected subjects in this study was 40.02 ± 1.23 years while that of the control was 35.04 ± 1.19 years. Forty six percent (46%; *n* = 46) and 37.5% (*n* = 30) of the HIV-infected subjects and the controls, respectively, were males while 54% (*n* = 54) and 62.50% (*n* = 50) of the HIV-infected subjects and controls, respectively, were females. Sixty percent (60%; *n* = 60) of the HIV-infected subjects were on ART while 40% were ART naïve. Nine percent (9%; *n* = 9) of the HIV-infected subject had CD4^+^ T-Cell count of < 200 cells/mm^3^. Of the 100 HIV-infected subject, 74% (*n* = 74) were anemic as against the 41.25% (*n* = 33) anemia seen in the control subjects using World Health Organization (WHO) cut-off of 12 g/dL and 13 g/dL for females and males, respectively [24] (Table 1).

**Table 1:** Demographic and clinical data of the HIV infected subjects and healthy control

Parameter	HIV Subjects *N* = 100	Control *N* = 80
AGE	40.02 ± 1.23^a^	35.04 ± 1.19^a^
GENDER		
Male	46 (46.00)	30 (37.50)
Female	54 (54.00)	50 (62.50)
HIV SUBJECTS ON ART	60 (60.00)	
ART Naïve HIV SUBJECTS	40 (40.00)	
CD4^+^T-CELL COUNT GROUP		
< 200 cells/mm^3^	9 (9.00)	0 (0.00)
≥ 200 cells/mm^3^	91 (91.00)	100 (100.00)
ANEMIA PREVALENCE	74 (74.00)^b^	33 (41.25)^b^

^a^mean + S.D

^b^cutoff according to WHO report 1968 [23]

The highest prevalence of anemia among the HIV-infected subjects was observed in the males (84.74%; 39/46) when compared with the female (64.81%; 35/54). Gender was significantly (*P* < 0.05) associated with anemia in HIV infection with an odd ratio (OR) of 1.958:1 (Male: Female [CI = 1.003–3.822). The ART naïve HIV-infected subjects had higher (90%; 36/40) prevalence of anemia when compared to the HIV-infected subjects on ART (63.33%; 38/60). ART status was significantly (*P* < 0.05) associated with anemia inn HIV infection with an OR of 0.607:1 (On ART: ART naïve [CI = 0.461–0.800). further stratification based on gender showed that males on ART treatment are more likely to be anemic than the females (OR = 2.026; CI = 0.967–4.247). There was no significant gender disparity among ART naïve subjects (*P* > 0.05). A 100% anemia was observed in the category of HIV infected subjects with CD4^+^ T-Cell count of < 200 cell/mm^3^. CD4^+^ T-Cell status was found not significantly (*P* > 0.05) associated with anemia in HIV infection (Table 2).

**Table 2:** Variable associated with anemia in HIV

Parameter	N. anemic (%)	X^2^	*P*-value	Odd ratio	95%CI
GENDER					
Male	39 (84.78)	5.14	0.023*	1.958	1.003–3.882
Female	35 (64.81)			1	
ART CATEGORY					
On ART	38 (63.33)	8.870	0.003*	0.607	0.461–0.800
ART Naïve	36 (90.00)			1	
On ART CATEGORY					
Male	21 (77.78)	4.411	0.036*	2.026	0.967–4.247
Female	17 (51.52)			1	
ART NAÏVE CATEGORY					
Male	18 (94.74)	0.902	0.342	2.000	0.355–11.265
Female	18 (85.71)			1	
CD4 CATEGORY					
< 200 cells/mm^3^	9 (100)		0.107^a^	0.878	0.807–0.956
≥ 200 cells/mm^3^	65 (71.43)			1	

^a^*p* value for fisher exact test

*N* absolute numbers; *Hb* hemoglobin, *GSH* glutathione, *GPX* glutathione peroxidase; *WBC* b white blood cell count; *CD* cluster of differentiation

*: Significantly different

Table 3 shows antioxidant and some hematological parameters of HIV-infected subjects and the control subjects. All assessed parameters (GSH, GPX, CD4^+^ T-cell count, hemoglobin, total white blood cell count, and platelet count of the HIV-infected subject were found to be significantly (*P* < 0.05) reduced in the HIV-infected subjects when compared with the control subjects.

**Table 3:** Antioxidant and some hematological parameters of HIV-infected and control subjects

	Subjects		
Parameter	HIV Subjects *N* = 100 Mean ± S.D.	Control Subjects *N* = 80 Mean ± S.D	*p*-value
ANTIOXIDANTS			
GSH (*μ* g/mL)	3.76+ 1.87	6.00+ 2.94	< 0.01*
GPX (pg/mL)	123.15 + 62.05	228.17 ± 80.04	< 0.01*
HEMATOLOGY			
CD4 (cells /mm^3^)	414.23+ 236.24	689.66 + 283.31	< 0.01*
Hb (g/dl)	11.27 + 1.84	12.61 + 1.70	< 0.01*
WBC (× 10^9^/L)	3.69 + 1.04	5.23 + 2.06	< 0.01*
Platelet (× 10^9^/L)	177.83 ± 62.13	243.14 + 71.99	< 0.01*

*N* absolute number; *Hb* hemoglobin; *GSH* glutathione

*GPX* glutathione peroxidase; *WBC* white blood cells count

*CD* cluster of differentiation

*: Significantly different

Table 4 shows the antioxidant and some hematological parameters of the HIV-infected subjects on antiretroviral therapy (ART) and ART-naïve group. All assessed parameters (GSH, GPX, CD4^+^ T-cell count, hemoglobin, total white blood cell count and platelet count were found to be significantly (*P* < 0.05) reduced in the ART naïve group when compared with those on ART therapy.

**Table 4:** Antioxidant and some hematological parameters of HIV infected subject on ART and ART naïve subjects

	HIV Subjects		
Parameter	Subjects on ART *N* = 60 Mean ± S.D	ART naive Subjects *N* = 40 Mean ± S.D	*p*-value
ANTIOXIDANTS			
GSH (*μ* g/mL)	4.32+ 2.19	2.93+ 0.66	< 0.01*
GPX (pg/mL)	143.22 + 71.96	93.04 ± 19.78	< 0.01*
HEMATOLOGY			
CD4 (cells /mm^3^)	442.17+ 242.99	342.33 + 208.58	< 0.01*
Hb (g/dL)	11.70 + 1.99	10.63 + 1.37	< 0.01*
WBC (× 10^9^/L)	4.02 + 1.15	3.21 + 0.58	< 0.01*
sPlatelet (× 10^9^/L)	205.33 ± 62.90	136.57 + 30.06	< 0.01*

*N* absolute number; *Hb* hemoglobin; *GSH* glutathione;

*GPX* glutathione peroxidase; *WBC* white blood cells count

*CD* cluster of differentiation

*: Significantly different

Table 5 shows the antioxidant and some hematological parameters of the HIV-infected subjects based on their CD4 category. All assessed parameters (GSH, GPX, CD4^+^ T-cell count, hemoglobin, total white blood cell count and platelet count were found to be significantly (*P* < 0.05) reduced in the < 200 cells/mm^3^ category when compared with the >200cells/mm^3^.

**Table 5:** Antioxidant and some haematological parameters the HIV infected subjects based on CD4 category

Parameters	CD4 category		*p*-value
	CD4 < 200 cells/mm^3^ *N* = 9 Mean ± S.D	CD4 ≥ 200 cells/mm^3^ *N* = 91 Mean ± S.D	
ANTIOXIDANTS			
GSH (μg/mL)	2.57 ± 0.59	3.88 ± 1.91	< 0.01*
GPX (pg/mL)	89.57 ± 20.34	126.47 ± 63.83	< 0.01*
HEMATOLOGY			
CD4 (cells/mm^3^)	131.56 ± 73.21	442.19 ± 283.31	< 0.01*
Hb (g/dL)	8.62 ± 2.22	11.53 ± 1.58	< 0.01*
WBC (× 10^9^/L)	2.66 ± 0.51	3.80 ± 1.02	< 0.01*
Platelet (× 10^9^/L)	102.58 ± 17.07	185.27 ± 59.98	< 0.01*

*N* absolute number; *Hb* hemoglobin; *GSH* glutathione;

*GPX* glutathione peroxidase; *WBC* white blood cells count

*CD* cluster of differentiation

*: Significantly different

Table 6 shows the antioxidant and some hematological parameters of HIV-infected subjects based on gender. GPX, CD4^+^ T-cell count, hemoglobin, and platelet values were comparable (*P* > 0.05) in both gender. Only glutathione (GSH) and total white blood cell count were significantly reduced in the males when compared with the females.

**Table 6:** Antioxidant and some haematological parameters the HIV infected subjects based on gender

Parameters	Gender		*p*-value
	Male HIV subjects *N* = 46 Mean ± S.D	Female HIV subjects *N* = 54 Mean ± S.D	
ANTIOXIDANTS			
GSH (μg/mL)	3.33 ± 1.38	4.13 ± 2.16	0.030*
GPX (pg/mL)	110.45 ± 50.56	133.96 ± 69.00	0.053
HEMATOLOGY			
CD4 (cells/mm^3^)	366.24 ± 207.33	455.11 ± 253.08	0.060
Hb (g/dL)	11.33 ± 2.19	11.22 ± 1.50	0.766
WBC (× 10^9^/L)	3.41 ± 0.70	3.94 ± 1.21	0.009*
Platelet (× 10^9^/L)	166.74 ± 54.26	187.28 ± 67.18	0.100

*N* absolute number; *Hb* hemoglobin; *GSH* glutathione;

*GPX* glutathione peroxidase; *WBC* white blood cells count

*CD* cluster of differentiation

*: Significantly different

Table 7 shows antioxidant and some hematological parameters of HIV-infected subjects on ART treatment based on duration of treatment. All the parameters (GSH, GPX, CD4^+^ T-cell count, hemoglobin, total white blood cell count and platelets) assessed were comparable (*P* > 0.05) in all groups.

**Table 7:** Antioxidants and some hematological parameters of HIV infected subjects on ART treatment based on duration of treatment

	Duration of ART treatment (years)			
Parameter	0–4 *N* = 32 Mean ± S.D	5–8 *N* = 18 Mean ± S.D	9–12 *N* = 10 Mean ± S.D	*p*-value
ANTIOXIDANTS				
GSH (μg/mL)	4.60 ± 2.03	4.62 ± 2.62	2.82 ± 1.27	0.731
GPX (pg/mL)	137.21 ± 63.37	162.72 ± 91.33	127.37 ± 56.44	0.374
CD4 (cells/mm^3^)	460.06 ± 247.25	484.44 ± 227.05	428.80 ± 277.33	0.851
HEMATOLOGY				
Hb (g/L)	11.69 ± 1.71	11.73 ± 2.00	11.68 ± 2.89	0.482
WBC (× 10^9^/L)	3.90 ± 1.04	4.11 ± 1.35	4.06 ± 1.16	0.644
Platelet (× 10^9^/L)	194.97 ± 58.32	211.94 ± 69.03	226.66 ± 65.20	0.337

*N* absolute number; *Hb* hemoglobin; *GSH* glutathione

*GPX* glutathione peroxidase; *WBC* white blood cells count

*CD* cluster of differentiation

Table 8 shows antioxidant and some hematological parameter of HIV infected subjects based on age. The values of all parameters assessed (GSH, GPX, CD4^+^ T-cell count, haemoglobin and total white blood cell count) were comparable (*P* > 0.05) across all age brackets except for platelet that significantly (*P* < 0.05) differ across the groups. The extreme age brackets (18–28 years and 49–60 years) had comparable platelet values (same mean subset) while the 29–38 and the 39–48 years age bracket had comparable platelet values (same mean subset).

**Table 8:** Antioxidant and some hematological parameters of HIV-infected subjects based on age

	Age of HIV-infected subjects (years)				
Parameter	18–28 *N* = 21 Mean ± S.D	29–38 *N* = 23 Mean ± S.D	39–48 *N* = 24 Mean ± S.D	49–60 *N* = 32 Mean ± S.D	*p*-value
ANTIOXIDANTS					
GSH (μg/mL)	3.99 ± 1.97	3.78 ± 1.59	4.28 ± 2.52	3.22 ± 1.28	0.189
GPX (pg/mL)	110.40 ± 49.51	137.25 ± 69.91	139.57 ± 76.54	109.06 ± 47.24	0.141
CD4 (cells/mm^3^)	451.76 ± 260	488.74 ± 322.66	340.25 ± 163.24	395.13 ± 177.59	0.164
HEMATOLOGY					
Hb (g/dL)	11.80 ± 1.71	11.01 ± 2.25	16.46 ± 1.82	10.97 ± 1.59	0.352
WBC (×10^9^/L)	3.42 ± 0.72	3.88 ± 1.46	3.75 ± 0.85	3.69 ± 0.99	0.559
Platelet (× 10^9^/L)	170.43 ± 55.77^a^	191.50 ± 70.16^b^	181.50 ± 70.16 ^b^	166.44 ± 62.65 ^a^	0.040*

*N* absolute number; *Hb* hemoglobin; *GSH* glutathione; *: Significantly different; Means with the same superscript belongs to the same mean group and do not differ significantly from each other

*GPX* glutathione peroxidase; *WBC* white blood cells count

*CD* cluster of differentiation

## Discussion

In this study, we observed significantly (*p* < 0.05) lower glutathione level in the HIV-infected subjects when compared with the control group. This finding is consistent with previous reports [11, 25, 26] indicating higher oxidative stress. Multiple mechanisms may contribute to systemic reduction of GSH in HIV infection. It could be due to an increased usage associated with low synthesis, secondary to the decrease in the availability of substrates [13, 25, 27]. It is known that in pathologic conditions, the three amino acid precursors of GSH synthesis (Glutamine, glycine and cysteine) become essential [28]. Secondly, long term production of inflammatory cytokines (such as IL-1, IL-17 and TNF-α) leads to production of free radicals. In turn, these free radicals are mopped up by free GSH. This cycle of high production of free radicals and their subsequent mopping ends up with glutathione depletion [29]. In addition, HIV-infected individuals have been reported to have increased levels of TGF-β in their plasma [30]. Increased TGF-β inhibits the production of Glutamine-cysteine ligase catalytic (GCLC) subunit which reduces the production of molecules of GSH. More so, HIV infection on its own may play vital role through the production and release of HIV-TAT (trans-acting transcriptional activator), [31] since TAT blocks transcription of manganese superoxide dismutase [32], an enzyme that helps to prevent oxidative stress and as well markedly decrease the activity of glucose-6-phosphate dehydrogenase, [21, 33] a key enzyme in pathway that maintain GSH in its reduced state. Choi et al., [34] in an animal study demonstrated that TAT decrease the amount of GSH present in mice through modulation of GSH biosynthetic enzymes. More so, Morris et al., [29] postulated that elevated levels of interleukin 1 (IL-1) also promotes depletion of intracellular cysteine, which in turn reduces the production of new GSH molecules considering the fact that cysteine availability is rate limiting for the synthesis of glutathione. The tripeptide glutathione is synthesized in a 2-step process in which cysteine and glutamate are linked by glutamate cysteine ligase to form gamma-glutamylcysteine which in turn, then linked to glycine by glutathione synthase [35]. T - cell function and viability have been reported to be markedly impaired in GSH depleted cells [21]. The glutathione redox system performs a critical role in eliminating oxidative stress in the body of oxidation stress and restores homeostasis. To exert antioxidant role, glutathione (GSH) is converted to oxidized form; Oxidized glutathione (GSSG) by glutathione peroxidase (GPX). GSSG can be converted back to GSH by glutathione reductase (GSR) [29].

This study observed significantly reduced glutathione peroxidase (GPX) in the HIV-infected subjects. This observation is in consonance with earlier reports [19, 36, 37]. However, this finding is at variance with earlier report by Delmas-Beauvieux et al., [38] who reported higher GPX value in the HIV-infected group. We could not “pin point” the reason for this discordance, but we cannot neglect the fact that the subjects used for their study were selenium and vitamin A deficient and had CD4^+^ T-cell count less than 0.4 × 10^9^/L. This protocol may have affected the outcome. GPX plays an important role in the metabolism of reactive oxygen species (ROS) as a defense mechanism against oxidative damage by catalyzing the reduction of a variety of hydroperoxides via glutathione as the reducing substrate. Antioxidant enzyme levels are sensitive to oxidative stress and alterations usually leads to cell damage and weakened antioxidant defense [19].

This study showed that HIV infected subjects on ART had significantly elevated glutathione (GSH) and glutathione peroxidase (GPX) levels. Few studies have examined the effect of ART on oxidative stress parameters and these studies remains conflicting. Just like the result of this study, Aukrust and colleagues [15] reported improvement in glutathione redox-status following administration of highly active antiretroviral therapy (HAART), though without full normalization. HAART administration was reported to suppress spontaneous release of TNF-α in PMBC from HIV infected subjects. Glutathione supplementation has been reported to increases cell proliferation in vitro [15]. More so, Sundaram and colleagues [19] reported increase in GPX in HIV infected subjects on ART over time. However, contrary report showed that ART is associated with increased oxidative stress [38].

This study observed significantly reduced glutathione peroxidase in HIV-infected subjects with CD4^+^ T-cell count < 200 cells/mm^3^. This observation is consistent with earlier report by Herzenberg and colleagues [21] who reported lower GSH in HIV-infected subjects with CD4^+^ T-cell count of < 200cells/mm^3^. CD4 count < 200 cells/mm^3^ has been reported to predict decreased survival [21].

We observed significantly lower glutathione level in the HIV-infected males when compared with the females. Previous animal studies have shown sex specific differences in antioxidant capacities in various tissues [39–41]. Lower levels of the H_2_O_2_ and higher levels of GSH and GPX activities have been reported in female mitochondria which is postulated to confer protection from ROS-mediated damage [42, 43].

We observed significantly lower CD4^+^ T-cell count in the HIV-infected subjects when compared with the control subjects. This observation is in consonance with previous findings. Depletion of CD4^+^ T-cell in HIV infection has been well documented [37, 44, 45]. The central event in HIV infection is destruction of CD4^+^ T-cells leading to a compromised immune system [46].

This study observed significantly lower hematological parameters in the HIV-infected subjects when compared with the control. Hematological indices provide physiological information on the blood picture and the reticuloendothelial system [47]. The hemoglobin, total white blood cell count and platelet count were all significantly lowered in the HIV-infected subjects. This finding is similar to previous reports, [44, 48] except in Asemota et al., [44] where the platelet counts were comparable. Anemia prevalence of 74% was observed in this study using 12 g/dL and 13 g/dL cutoff for females and males, respectively. This value is similar to 80% obtained in another study in Nigeria [49]. However, lower values of 56% [44], 60.1% [50] and 64.0% [51] have been reported. The Anemia Prevalence Study Group [52], Women’s Interagency HIV study (WIHS) [53] and Human Immunodeficiency Virus Epidemiology Research (HER) [54] reported that HIV-positive patients who were African American demonstrated a higher prevalence in anemia than found in other races. This disparity in anemia prevalence may be due to regional differences. A common cause of anemia among patients living with HIV is blood loss. This might be associated with neoplastic conditions such as Kaposi sarcoma in the gastrointestinal tract or gastrointestinal injury that follow opportunistic cytomegalovirus infection. Apart from blood loss, the pathophysiology of HIV involving anemia may be explained via three fundamental mechanisms which are: elevated red blood cell destruction, reduction in red blood cell production and ineffective red blood cell production [55]. More so, Zidovudin treatment has been reported to induce bone marrow suppression and consequently increase odd of developing anemia [56–58]. Retrospective analysis has shown that baseline anemia is correlated with decreased survival, and increased disease progression in HIV infection [50, 51]. Unlike the result of this study, the Anemia Prevalence Study Group [52] reported 71% greater prevalence of anemia among women than among men in America using cutoff point of 12 g/dL and 13 g/dL for females and males respectively. However, an earlier study in Nigeria by Omoregie and colleagues [50] reported no significant disparity in anemia among HIV-infected subjects based on gender, but on further stratification, reported significantly higher anemia prevalence among ART naïve males than their female counterpart. We observed that majority of the ART naïve HIV-infected subjects were anemic in relation to HIV infected subjects on ART. This goes further to corroborate earlier submission of the ameliorative effect of ART on anemia status in HIV infection [44]. Further stratification of the HIV-infected subjects on ART based on gender showed that males on ART treatment had significantly higher prevalence of anemia than their female counterpart. This trend may partly explain the reason of the gender disparity on anemia status observed in this study. Males have been reported to have poor adherence to therapy than females. A report from Malawi [59] showed that men have lower compliance to therapy as indicated by their higher loss to follow-up (18.6% versus 13.0%). Furthermore, men have been reported to initiate ART late in HIV infection than women. Prevention of mother to child transmission has been proposed as an explanation for early detection and subsequent presentation for ART [60]. More so, we observed higher odds of anemia in HIV infection in those with CD4^+^ T-cell count < 200 cells/mm^3^. This corroborates the fact that CD4^+^ T-cells are protective of the cytotoxic pathology of the HIV infection.

## Conclusions

This study found significant changes in antioxidants such as GSH and GPX which was more critical in the ART naïve HIV-infected subjects. Supplementation with glutathione or antioxidants may improve the condition. Some authors have reported improved survival associated with oral N-acetylcystein (NAC) administration in HIV infection [61, 62].

## Data Availability

Datasets generated and analysed in this study are available from the corresponding author on request.

## Abbreviations

3TC: Lamivudine
ABC: Abacavir
ART: Antiretroviral therapy
ATV: Atazanavir
AZT: Zidovudine
CD: Cluster of differentiation
DRV: Darunavir
DTG: Dolutegravir
EFV: Efavirenz
ELISA: Enzyme-linked immunosorbent assay
FTC: Emtricitabine
GCL: Glutathione-cysteine ligase
GPX: Glutathione peroxidase
GSH: Glutathione
GSR: Glutathione reductase
GSS: Glutathione synthase
GSSG: Oxidised glutathione
HIV: Human immunodeficiency virus
HRP: Horseradish peroxidase
IL: Interleukin
INI: Integrase inhibitor
LPV: Iopinavir
NADPH: Nicotinamide adenine dinucleotide phosphate
NF: Nuclear factor
NNRTI: Non-nucleoside reverse transcriptase inhibitor
NRTI: Nucleoside/nucleotide reverse transcriptase inhibitor
NVP: Nevirapin
OR: Odd ratio
PI: Protease inhibitor
r: Ritonavir
RAL: Raltegravir
rGSH: Reduced glutathione
RNA: Ribonucleic acid
TAT: Trans-acting transcriptional activator
TDF: Tenofovir disoproxil fumarate
TMB: Tetramethylbenzydine
WBC: White blood cell
WHO: World health organization
